# Relative facial width, and its association with canine size and body mass among chimpanzees and bonobos: Implications for understanding facial width‐to‐height ratio expression among human populations

**DOI:** 10.1002/ajpa.25040

**Published:** 2024-11-11

**Authors:** Katharine L. Balolia, Kieran Baughan, Jason S. Massey

**Affiliations:** ^1^ School of Archaeology and Anthropology Australian National University Canberra Australian Capital Territory Australia; ^2^ Department of Anthropology Durham University Durham UK; ^3^ Department of Anatomy and Developmental Biology Monash University Melbourne Victoria Australia

**Keywords:** apes, facial width‐to‐height ratio, morphometrics, sexual selection, skull

## Abstract

**Objectives:**

Facial width‐to‐height ratio (fWHR) has been widely investigated in the context of its role in visual communication, though there is a lack of consensus about how fWHR serves as a social signal. To better understand fWHR variation in a comparative context, we investigate the associations between fWHR and canine crown height (CCH) and body mass, respectively, among two chimpanzee subspecies (*Pan troglodytes schweinfurthii*, *Pan troglodytes troglodytes*) and bonobos (*Pan paniscus*).

**Materials and Methods:**

We collected landmark data from 3D surface models of 86 *Pan* cranial specimens to quantify fWHR and upper CCH, and to estimate body mass. We used Spearman's *r* and Kruskal‐Wallis tests to test for significant relationships among variables, and to assess sexual dimorphism.

**Results:**

There is an inverse relationship between fWHR and CCH in both sexes of *Pan*, however there are interpopulation differences in the relationship between fWHR and CCH among *Pan* taxa. *Pan paniscus* have relatively wide faces and small canine crowns, and wide faces in *Pan t. schweinfurthii* males may be driven by body size constraints. *Pan troglodytes* and *Pan paniscus* show fWHR dimorphism, and *Pan paniscus* have significantly higher fWHRs than do either *Pan troglodytes* subspecies.

**Discussion:**

Our findings indicate that CCH and facial breadth may serve subtly different signaling functions among *Pan* taxa. Further research into the circumstances in which wide faces evolved among chimpanzees and bonobos will likely afford deeper insights into the function of relatively wide faces in the context of visual signaling among humans and our extinct hominin relatives.

## 1 INTRODUCTION

Static and dynamic features of facial morphology serve as important visual signals in humans and non‐human primates alike, communicating information about sex, identity and perceived indicators of personality and behavioral tendencies (Andrew, 1963; Dixson et al., 2005; Geniole et al., 2015; Gerald, 2003; Jack & Schyns, 2015; Lefevre, Etchells, et al., 2014; Parr, 2011; Petersen & Higham, 2020; Rhodes, 2006; Waller et al., 2022; Wilson & Masilkova, 2023). Among humans, much research to date has specifically sought to understand whether relative facial width, often measured as the facial width‐to‐height ratio (fWHR), serves as a visual signal of personality traits and exhibited behaviors, including perceived dominance behavior (associated with attempts to control others, or to get one's own way), aggressive behavior, threatening behavior, fighting ability, formidability, co‐operative behavior and trustworthiness (Geniole et al., 2015; Haselhuhn et al., 2015; Sell et al., 2009; Stirrat & Perrett, 2010; Zilioli et al., 2015). However, the claim that relative facial width reliably serves a social signaling function is not universally supported. Early research on this topic suggests that facial metrics are sound predictors of aggressive behavior (Carré et al., 2009; Carré & McCormick, 2008), and subsequent research has shown statistical associations between fWHR and traits such as fighting ability, upper body strength, formidability, self‐ and other‐perceived dominance behavior, psychopathic traits, trustworthiness, integrity, cues of threat, physical aggression, judgments of aggressiveness and post‐conflict resolution judgments (Sell et al., 2009; Stirrat & Perrett, 2010; Alrajih & Ward, 2014; Lefevre, Etchells, et al., 2014; Mileva et al., 2014; Zilioli et al., 2015; Anderl et al., 2016; Ormiston et al., 2017; MacDonell et al., 2018; Yang et al., 2018; Kajonius & Eldblom, 2020; Wen & Zheng, 2020; Merlhiot et al., 2021; Brown et al., 2022; Caton, Hannan, & Dixson, 2022; Caton, Pearson, & Dixson, 2022; Caton et al., 2024). A smaller number of studies however have argued that there is weak support for an association between relative facial breadth and aggression as well as other behavioral tendencies and personality traits, where these authors provide evidence to show that in a modern context, greater fWHR values are associated with biased perceptions of negative characteristics rather than antisocial or undesirable behaviors, including aggressive and power‐mediated behavior (Gómez‐Valdés et al., 2013; Kosinski, 2017; Wang et al., 2019). Some of these authors argue that a potential mismatch in the relationship between fWHR and behavior may be prevalent in a modern context among humans, compared to the function of relative facial width in an ancestral context (Durkee & Ayers, 2021; Wang et al., 2019). More specifically, these authors argue that relatively wide faces are not reliably associated with antisocial tendencies (Wang et al., 2019), though in a modern context they continue to be associated with biased perceptions of aggressiveness, meanness and antisocial tendencies (Durkee & Ayers, 2021).

Modern human populations vary in their sex‐specific expression of fWHR and there is evidence to suggest that this is a sexually selected trait, at least among some populations. Among Australian citizens of mixed ethnicity, fWHR sexual dimorphism was only found in young adulthood, where young adult males show larger fWHR values compared to older adult males (Summersby et al., 2022). Black South Africans show sex differences in growth trajectories for bizygomatic breadth, which are not observed for facial height (Weston et al., 2007). Similarly, research among a population of US army personnel shows that bizygomatic breadth is highly sexually dimorphic, a relationship which remains significant when adjusting for facial height and other craniometric and body size measurements (Caton & Dixson, 2022). However, male‐biased sexual dimorphism for relative facial breadth is not universal among populations, where among Buryats of Southern Siberia and Caucasian young adults, females show significantly higher fWHRs than do males (Lefevre et al., 2012; Rostovtseva et al., 2021). Other studies show that among Africans, a Turkish sample and other European populations, there is no evidence for fWHR sexual dimorphism (Kramer et al., 2012; Lefevre et al., 2012; Özener, 2012). Meta‐analyses show small, significant male‐biased fWHR sexual dimorphism when 87 samples were combined (Kramer, 2017). However, the validity of an approach that seeks to lump together many populations to detect fWHR sexual dimorphism among humans may be questioned given that individual human populations are known to vary in the presence and pattern of sex‐ and age‐specific fWHR expression, as detailed above.

Among non‐human primates, while there are fewer studies that have sought to investigate the relationship between behavioral tendencies or personality traits and fWHR, evidence suggests that fWHR is a sexually selected trait among some non‐human primates (Borgi & Majolo, 2016; Lefevre, Wilson, et al., 2014; Wilson et al., 2014; Wilson et al., 2020). In a broader comparative context, understanding the nature of associations between fWHR and behavioral and personality traits is likely to be informative in understanding how facial signaling is associated with primate socioecology and associated selective pressures (Wilson & Masilkova, 2023). Among capuchin monkeys, macaques, chimpanzees and bonobos, relatively wide faces are often associated with alpha status (the highest‐ranking individual, assessed by behavioral observations), affiliative dominance (i.e., assertiveness, including independent, decisive and persistent behavior), agonistic dominance (typified by individuals who elicit a high proportion of fleeing behavior during agonistic encounters), female dominance style (ranked classification of grades 1–4 based on social tolerance and conciliatory tendencies, following Thierry, 2000), personality dominance, confidence and assertiveness (each measured as personality traits, using a 54 item Hominoid Personality Questionnaire (HPQ), following Weiss et al., 2009) (Altschul et al., 2019; Borgi & Majolo, 2016; Lefevre, Wilson, et al., 2014; Martin et al., 2019; Wilson et al., 2014; Wilson et al., 2020). Among brown capuchin monkeys (*Cebus [Sapajus] apella*), fWHR is associated with assertiveness and alpha status among males, with age and sex being significantly associated with fWHR, and where fWHR sexual dimorphism is observed in adults, but not juveniles (Lefevre, Wilson, et al., 2014; Wilson et al., 2014). Age is also significantly associated with higher fWHRs, and alpha status individuals have larger fWHRs, even when controlling for body weight (Lefevre, Wilson, et al., 2014). Among macaques (*Macaca sp*.), a similar pattern is found, where higher fWHRs are observed in despotic species, compared to tolerant ones (Borgi & Majolo, 2016), and female rhesus macaques (*Macaca mulatta*) bias their visual attention towards male macaque faces with higher fWHRs (Rosenfield et al., 2019). Other authors have shown that among rhesus macaques (*Macaca mulatta*), assertiveness is positively associated with fWHR, though only in younger individuals (<8 years old) (Altschul et al., 2019). Chimpanzees and bonobos (*Pan troglodytes* and *Pan paniscus*) vary in how personality and behavioral traits are associated with fWHR. Among chimpanzees, only *Pan t. verus* females show a positive association between fWHR and dominance as a personality variable (HPQ Personality rating), whereas no relationship between fWHR and five other personality components (extraversion, conscientiousness, agreeableness, neuroticism and openness) is observed in *Pan t. verus* males, or in either sex of *Pan t. schweinfurthii* or *Pan t. troglodytes* (Wilson et al., 2020). These authors found that age was not significantly associated with fWHR among *Pan troglodytes* (Wilson et al., 2020). In bonobos, agonistic dominance and affiliative dominance are strong predictors of fWHR in males and females, independent of the effects of age and body weight (Martin et al., 2019). Facial width‐to‐height ratio sexual dimorphism is observed among bonobos (*Pan paniscus*) (Martin et al., 2019), with male fWHR exceeding female fWHR. No sexual dimorphism is found in three chimpanzee subspecies (*Pan t. verus*, *Pan t. schweinfurthii* or *Pan t. troglodytes*) (Wilson et al., 2020). Female dominance style, dominance as a personality trait, affiliative dominance or agonistic dominance may also drive high fWHRs among some macaque species (*Macaca* spp.), western chimpanzees (*Pan troglodytes verus*) and bonobos (*Pan paniscus*) (Borgi & Majolo, 2016; Martin et al., 2019; Wilson et al., 2020). The relationship between measures of dominance and fWHR among females exists either in conjunction with, or independent of, the relationship between dominance and fWHR in males (Borgi & Majolo, 2016; Martin et al., 2019; Wilson et al., 2020).

Combined, the available human and non‐human primate research into the underlying basis of fWHR expression shows a lack of clarity and consensus surrounding the role that fWHR plays as a visual indicator of aspects of behavior and personality. There is also a paucity in our understanding of the causal factors underlying variation in fWHR expression, and a greater understanding of the relationship between fWHR and other morphological variables among our closest living relatives, the chimpanzees and bonobos, will provide a deeper context to better understand the role of relatively wide faces as a signal of aggression, dominance and other aspects of personality and behavior within a broader comparative context. Avenues of enquiry that may allow a better understanding of the underlying basis for variation in fWHR among *Pan* taxa include investigations into the relationships between fWHR and other morphological variables, including canine crown height (CCH), and body weight or size. Research suggests that interspecific variation in fWHR may be associated with CCH, for which a negative association between facial breadth dimorphism and CCH dimorphism has been shown among anthropoids (Weston et al., 2004). Findings by Weston et al. (2004) indicate that wider faces are associated with reduced CCH, where the authors suggest that among taxa with relatively small canines, facial structure may serve as a replacement of canine size in the context of visual signaling. This is in the context of the well‐documented role that large canine teeth play in male and female primates, where canine teeth serve as a socially or sexually selected trait, used for visual signaling and weaponry, associated with mating system, intensity of inter‐male competition, and the operational and socionomic sex ratio among non‐human primates (Leutenegger & Kelly, 1977; Plavcan, 1998; Plavcan, 2004; Plavcan et al., 1995; Plavcan & van Schaik, 1992, 1993). Canine crown height can also play a vital role in the avoidance of conflict escalation given the high costs of high‐intensity combat encounters among males (Setchell & Wickings, 2005). Other research, discussed above, suggests that there is a relationship between fWHR and body weight in some primate species. As previously noted, among bonobos, affiliative dominance and agonistic dominance are associated with fWHR, though this is independent of the influence of body weight (Martin et al., 2019). Similarly, among brown capuchin monkeys, the relationship between alpha status and fWHR is independent of body weight, though in this species there is a positive association between fWHR and body weight (Lefevre, Wilson, et al., 2014). Based on these findings, a relationship between fWHR and body weight is expected among some primate species, though how and why these relationships may differ among taxa is currently unclear.

To date, no research has been conducted to investigate whether there is an inverse relationship between fWHR and CCH among *Pan* males and females at the species‐ or population levels, or to understand how body mass covaries with fWHR. Furthermore, the underlying reasons for interspecific differences in fWHR expression are yet to be fully understood. In the research presented here, we test Weston et al.'s (2004) hypothesis that fWHR is negatively correlated with upper CCH in three *Pan* taxa (*Pan troglodytes schweinfurthii*, *Pan troglodytes troglodytes* and *Pan paniscus*). If relative facial breadth (i.e., relatively wide faces) serves as a replacement of canine size in the context of visual signaling, we expect to find an inverse relationship between these two variables given the role of canine crowns as a visual threat of aggression (Leutenegger & Kelly, 1977; Plavcan, 1998; Plavcan, 2004; Plavcan et al., 1995; Plavcan & van Schaik, 1992, 1993; Setchell & Wickings, 2005). We further test for fWHR and CCH sexual dimorphism in each *Pan* group. We evaluate whether larger fWHRs show a statistical association with body mass estimates, though we make no prediction about this association given the paucity of previous research on this topic. We do not examine the relationship between CCH and body mass in this study as our main aim in conducting this research is to understand variation and the morphological correlates of relative facial width among chimpanzees and bonobos. We test for intergroup differences in fWHR to understand whether there is increased emphasis on relatively wide faces among any of the three chimpanzee and bonobo groups investigated as part of this study in the context of patterns of sexual dimorphism observed among *Pan*.

## 2 MATERIALS AND METHODS

### 2.1 Study sample

The sample consists of 86 dentally mature wild‐shot cranial specimens of Eastern chimpanzees (*Pan t. schweinfurthii*; 30 specimens), Central chimpanzees (*Pan t. troglodytes*; 30 specimens) and bonobos (*Pan paniscus*; 26 specimens). We define dental maturity as M^3^s being in full occlusion (Balolia et al., 2013). We further selected specimens on the basis that canines were fully erupted and exhibited low amounts of wear. Males and females are represented equally in the sample (Table 1).

**TABLE 1:** Geographical locations, repositories and sex breakdown of the *Pan* specimens used in the present study.

Taxon	Males	Females	Geographical locations	Repository
*Pan troglodytes schweinfurthii*	15	15	Specimens were sampled from a wide range of localities across the Democratic Republic of Congo (DRC)	Royal Museum of Central Africa, Tervuren, Belgium
*Pan troglodytes troglodytes*	15	15	Abong Mbong (French Cameroon) Batouri, Ebolwa (Cameroon)	Cleveland Museum of Natural History, Ohio, USA; Powell Cotton Museum, Kent, UK
*Pan paniscus*	11	15	Ilima, Ubundu (DRC)	Royal Museum of Central Africa, Tervuren, Belgium

### 2.2 Data collection

**FIGURE 1:**
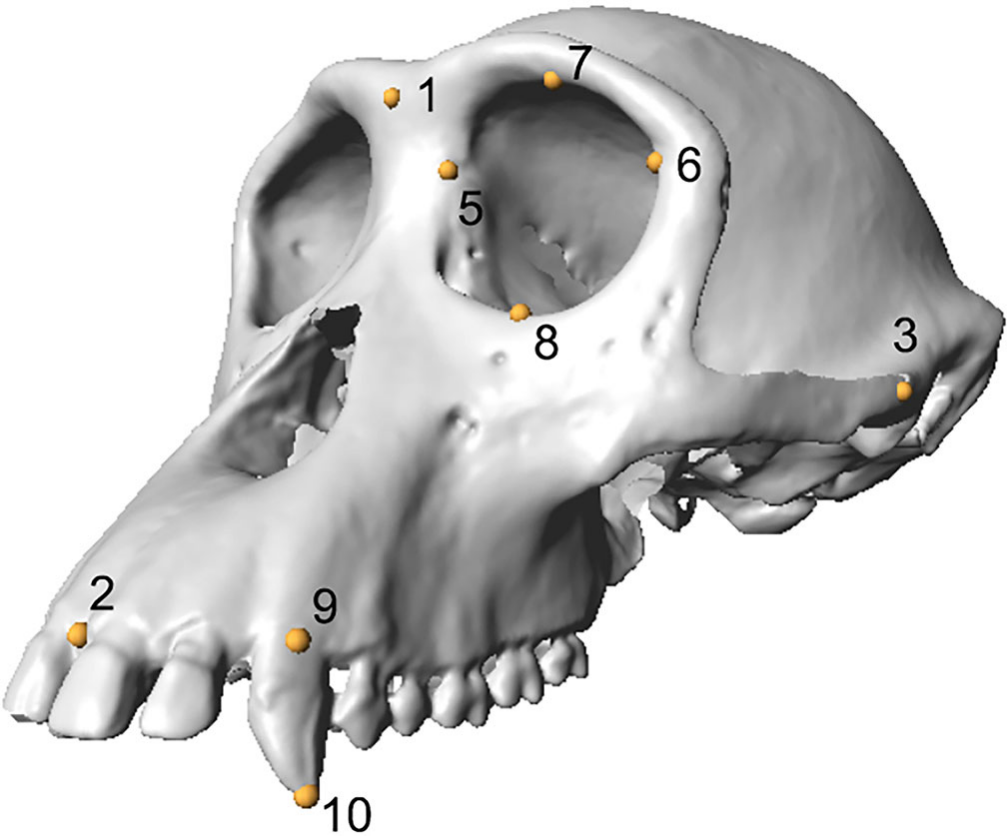
3D landmarks used to quantify facial width‐to‐height ratio (fWHR), orbital breadth, orbital height and canine crown height (CCH), applied to a *Pan troglodytes troglodytes* 3D surface model. Bilateral landmarks are depicted on the left side only. Landmarks are defined in Table 2.

Landmark data were collected from 3D surface models, which were collected using a Breuckmann SmartSCAN white light scanner. For each specimen we collected four landmarks to quantify fWHR, four landmarks on the orbital margin to estimate body mass and two landmarks to quantify upper CCH (Figure 1, Table 2).

**TABLE 2:** 3D landmarks used to quantify facial width‐to‐height ratio (fWHR), orbital breadth, orbital height and canine crown height (CCH) of the *Pan* specimens used in this study.

LM#	Definition	Associated measurement
1	*Glabella*: the most anterior projecting midline point of the frontal bone	Facial height
2	*Prosthion*: the most anterior projecting midline point on the alveolar margin between the central maxillary incisors	Facial height
3	*Zygion* (left): most lateral extent of the lateral aspect of the zygomatic arch	Bizygomatic breadth (facial width)
4	*Zygion* (right): most lateral extent of the lateral aspect of the zygomatic arch	Bizygomatic breadth (facial width)
5	*Maxillofrontale*: point where the anterior lacrimal crest of the maxilla meets the frontomaxillary suture	Orbital breadth
6	Ectoconchion: point at the most lateral aspect of the orbital margin	Orbital breadth
7	Upper margin of the orbital cavity	Orbital height
8	Lower margin of the orbital cavity	Orbital height
9	Midpoint of the labial surface at the alveolar margin on the canine crown	Canine crown height (CCH)
10	Most inferior point of the canine crown	Canine crown height (CCH)

*Note*: Landmarks (LMs) are depicted in Figure 1.

We quantified fWHR as bizygomatic breadth (distance between the left and right zygion)/facial height (distance between glabella and prosthion). To estimate body mass, we calculated an orbital area as an ellipse, a strong predictor of body mass in hominoids with a correlation coefficient of 0.99 (Spocter & Manger, 2007). We calculated this variable as orbital breadth (minimum distance between maxillofrontale and ectoconchion) × orbital height (minimum distance between upper and lower margins of the orbital cavity, taken at a right angle to orbital breadth) × π. This was taken from the left side of each specimen (Spocter & Manger, 2007) (Figure 1, Table 2). We measured upper CCH, quantified as the distance between the midpoint of the labial surface at the alveolar margin on the canine crown and the most inferior point of the canine crown (Table 2). We used the maximum upper CCH value (i.e., the tooth exhibiting the least amount of dental wear) for each specimen in our analysis.

We measured fWHR and CCH sexual dimorphism by calculating the index of sexual dimorphism (ISD = mean male value/mean female value). We collected 3D landmarks and associated measurements using Stratovan Checkpoint v. 2017.03.03.0771.

### 2.3 Data analysis

We used Spearman's *r* (Spearman, 1904) to test for statistically significant relationships between variables (i.e., fWHR, CCH and body mass estimates). We tested for fWHR and CCH dimorphism, and for interspecific differences in fWHR estimates using independent‐samples Kruskal‐Wallis tests (Kruskal & Wallis, 1952). For all analyses, we used a *p* ≤ 0.05 threshold to assess statistical significance. We performed Spearman's *r* analyses (denoted by *r*_*s*_) and Kruskal‐Wallis tests using SPSS v. 28.

### 2.4 Ethical note

No ethical clearance was required for this study.

## 3 RESULTS

**FIGURE 2:**
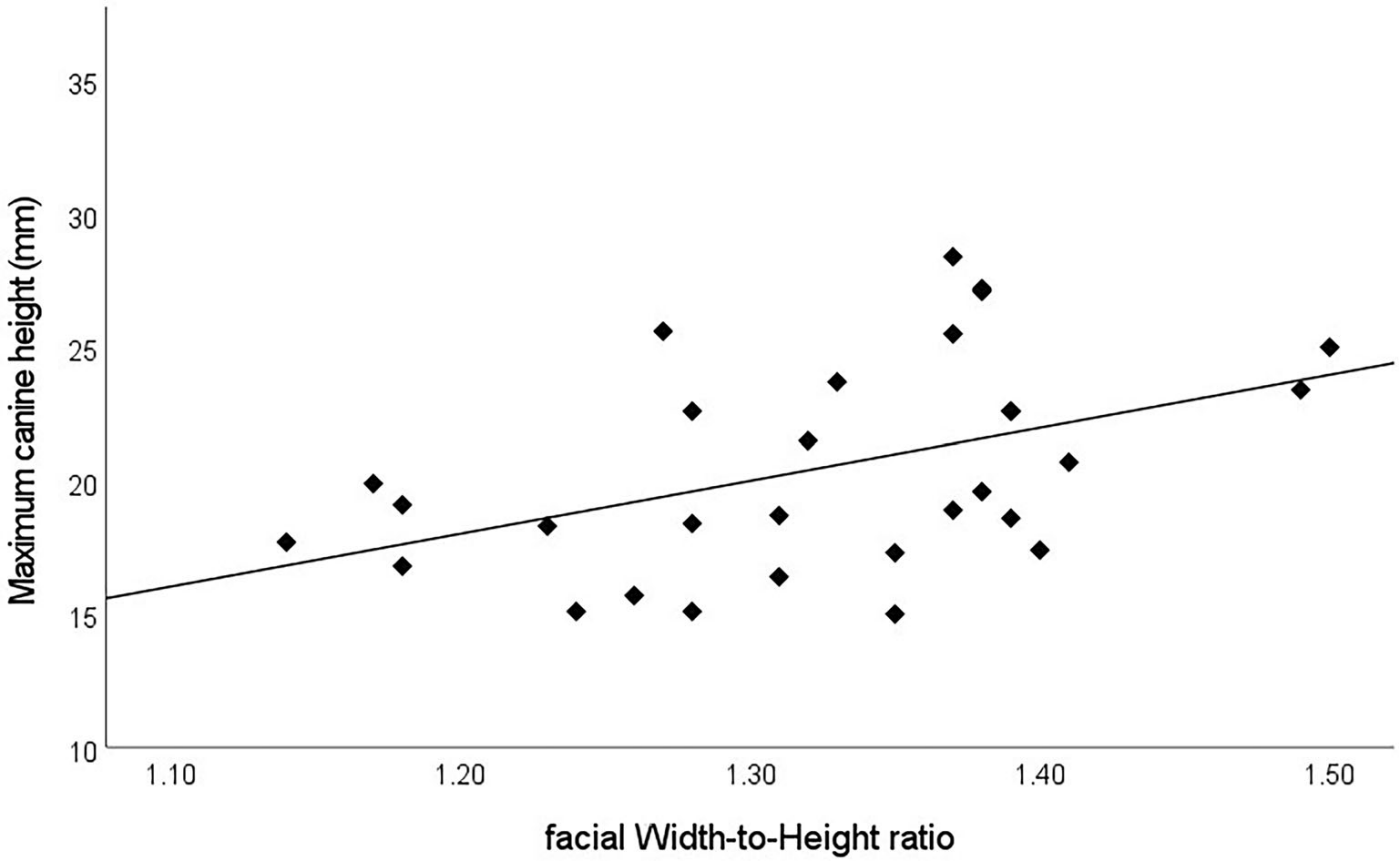
Facial width‐to‐height ratio (fWHR) and canine crown height (CCH) regression in *Pan troglodytes troglodytes* (R^2^ = 0.203, f _(1,29)_ = 7.119, *p* = 0.013).

**FIGURE 3:**
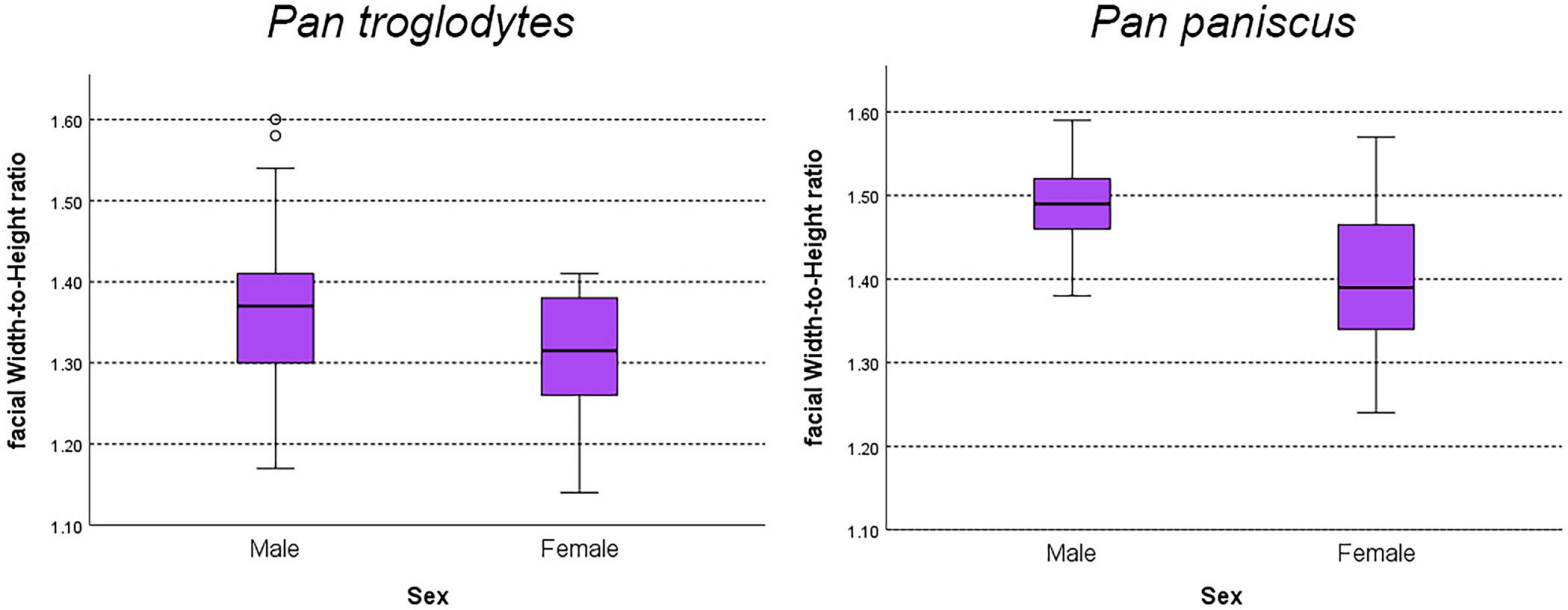
Boxplots of sex‐specific facial width‐to‐height (fWHR) ratio values for *Pan troglodytes* and *Pan paniscus*, who both show statistically significant fWHR sex differences (*p* < 0.05). Sexual dimorphism values, associated sex‐specific summary statistics and statistical test results are provided in Table 4.

**FIGURE 4:**
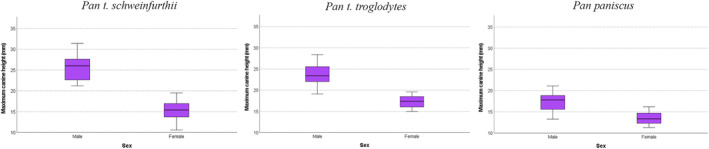
Boxplots of sex‐specific canine crown height (CCH) values for *Pan troglodytes schweinfurthii*, *Pan troglodytes troglodytes* and *Pan paniscus*. All three taxa show statistically significant CCH sex differences (*p* < 0.001). Sexual dimorphism values, associated sex‐specific summary statistics and statistical test results are presented in Table 5.

Consistent with our predictions, there is an inverse relationship between fWHR and CCH in *Pan* (males: *r*_*s*_ = −0.337, *n* = 39, *p* = 0.036; females: *r*_*s*_ = −0.384, *n* = 44, *p* = 0.01). Post‐hoc tests reveal that among males, this result is driven by *Pan paniscus* males, who show an inverse correlation between fWHR and CCH (*r*_*s*_ = −0.667, *n* = 9, *p* = 0.05). No other group shows a significant association between fWHR and CCH when the sample is broken down by taxon and sex (Table 3). *Pan t. troglodytes* show a positive correlation between fWHR and CCH (*r*_*s*_ = 0.432, *n* = 30, *p* = 0.017) (Figure 2), and sex‐specific analyses show fWHR sexual size dimorphism among *Pan troglodytes* and *Pan paniscus*, where *Pan troglodytes* male fWHR is 5% larger than female fWHR, and *Pan paniscus* male fWHR is 6% larger than female fWHR (Table 4, Figure 3). No fWHR sex differences are found in *Pan troglodytes* at the subspecies level, likely due to a lack of statistical power (*Pan t. schweinfurthii*: H_(1)_ = 2.297, *p* = 0.130; *Pan t. troglodytes*: H_(1)_ = 2.628, *p* = 0.105). All three *Pan* taxa show CCH sexual dimorphism (*Pan t. schweinfurthii* ISD = 1.66; *Pan t. troglodytes* ISD = 1.37; *Pan paniscus* ISD = 1.29; Table 5, Figure 4).

**TABLE 3:** Facial width‐to‐height ratio (fWHR) and canine crown height (CCH) by sex for *Pan troglodytes schweinfurthii*, *Pan troglodytes troglodytes* and *Pan paniscus*.

Taxon/sex	Spearman's *r*
*P. t. schweinfurthii* males	*r*_*s*_ = −0.128, *n* = 15, *p* = 0.649, ns
*P. t. schweinfurthii* females	*r*_*s*_ = −0.306, *n* = 15, *p* = 0.268, ns
*P. t. troglodytes* males	*r*_*s*_ = 0.246, *n* = 15, *p* = 0.376, ns
*P. t. troglodytes* females	*r*_*s*_ = 0.347, *n* = 15, *p* = 0.205, ns
*P. paniscus* males	***r***_***s***_ **= −0.667, *n* = 9, *p* = 0.05**
*P. paniscus* females	*r*_*s*_ = −0.424, *n* = 14, *p* = 0.131, ns

*Note*: Results presented in bold are statistically significant.

Abbreviation: ns, not significant.

**TABLE 4:** Facial width‐to‐height ratio (fWHR) sexual dimorphism for *Pan troglodytes* and *Pan paniscus*.

Taxon/sex	*N*	Mean	SD	ISD	Kruskal‐Wallis test
*P. troglodytes* males	30	1.37	0.104	**1.05**	**H**_**(1)**_ **= 4.513, *p* = 0.034**
*P. troglodytes* females	30	1.31	0.076		
*Pan paniscus* males	9	1.49	0.061	**1.06**	**H**_**(1)**_ **= 5.845, *p* = 0.016**
*Pan paniscus* females	15	1.40	0.089		

Abbreviations: SD, standard deviation; ISD, index of sexual dimorphism.

**TABLE 5:** Canine crown height (CCH) sexual dimorphism for *Pan troglodytes schweinfurthii*, *Pan troglodytes troglodytes* and *Pan paniscus*.

Taxon/sex	*N*	Mean	SD	ISD	Kruskal‐Wallis test
*P. t. schweinfurthii* males	15	25.37	3.27	**1.66**	**H**_**(1)**_ **= 21.784, *p* < 0.001**
*P. t. schweinfurthii* females	15	15.25	2.33		
*P. t. troglodytes* males	15	23.66	2.77	**1.37**	**H**_**(1)**_ **= 21.413, *p* < 0.001**
*P. t. troglodytes* females	15	17.27	1.52		
*Pan paniscus* males	11	17.30	2.36	**1.29**	**H**_**(1)**_ **= 12.094, *p* < 0.001**
*Pan paniscus* females	14	13.43	1.46		

*Note*: Results presented in bold are statistically significant.

Abbreviations: SD, standard deviation; ISD, index of sexual dimorphism.

**FIGURE 5:**
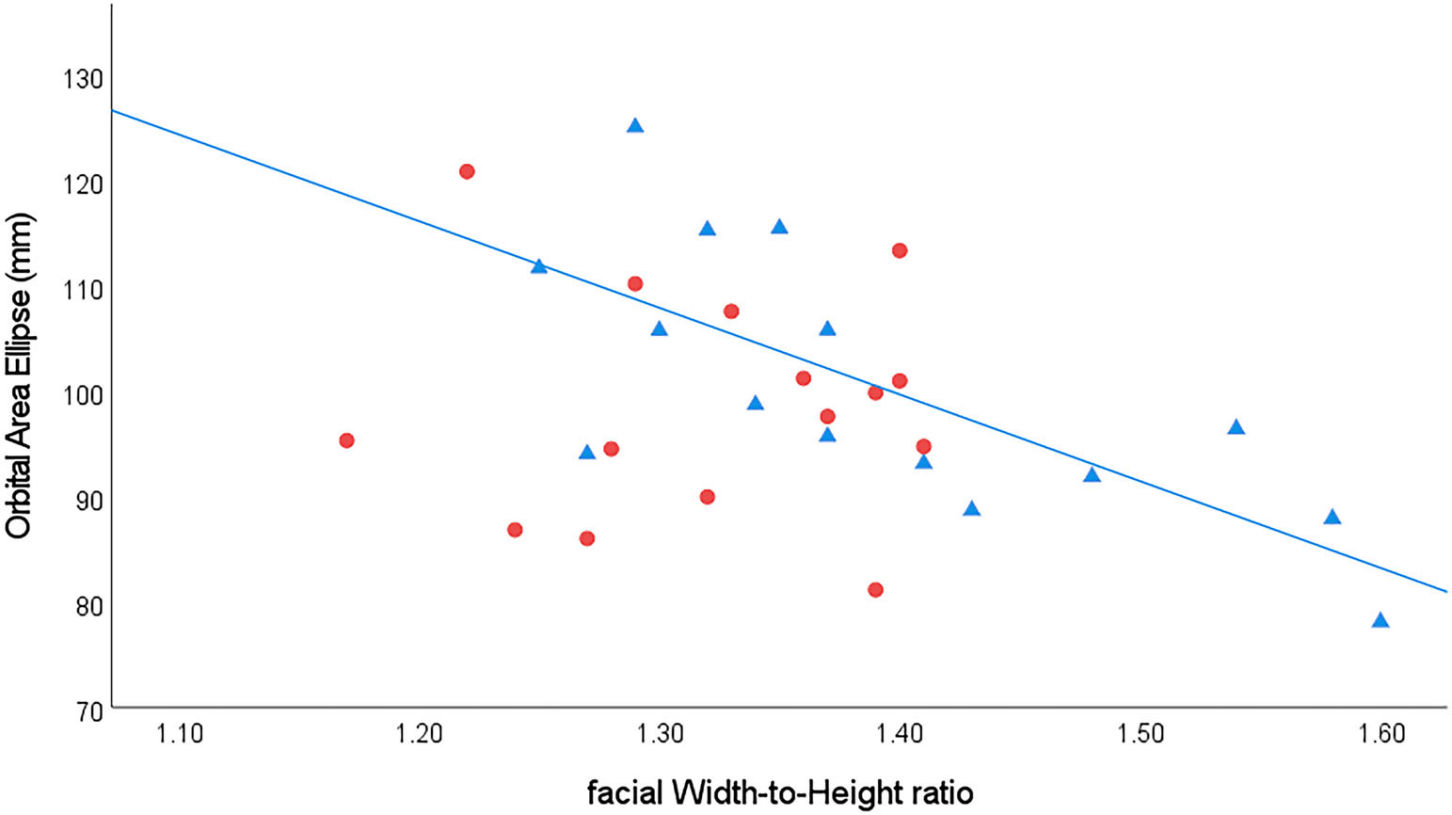
Facial width‐to‐height ratio (fWHR) and orbital area ellipse regressions (proxy for body mass) for *Pan troglodytes schweinfurthii*. Males = blue triangles and associated regression line (R^2^ = 0.528, f_(1,14)_ = 14.558, *p* = 0.002), females = red circles (R^2^ = 0.028, f_(1,14)_ = 0.010, *p* = 0.922, not significant).

Analyses to evaluate whether larger fWHRs are associated with body mass estimates show a strong inverse relationship between fWHR and body mass in *Pan t. schweinfurthii* males (*r*_*s*_ = −0.726, *n* = 15, *p* = 0.002) (Table 6, Figure 5). No similar effect is found in *Pan t. schweinfurthii* females (*r*_*s*_ = 0.104, *n* = 15, *p* = 0.713), or in the males or females of any other *Pan* taxon (Table 6). These findings suggest that *Pan t. schweinfurthii* body size constraints may be driving increased fWHRs among males, but not in females.

**TABLE 6:** Spearman's *r* results between facial width‐to‐height ratio (fWHR) and body mass estimates by sex for *Pan troglodytes schweinfurthii*, *Pan troglodytes troglodytes* and *Pan paniscus*.

Taxon/sex	Spearman's *r*
*P. t. schweinfurthii* males	***r***_***s***_ **= −0.726, *n* = 15, *p* = 0.002**
*P. t. schweinfurthii* females	*r*_*s*_ = 0.104, *n* = 15, *p* = 0.713, ns
*P. t. troglodytes* males	*r*_*s*_ = −0.315, *n* = 15, *p* = 0.253, ns
*P. t. troglodytes* females	*r*_*s*_ = −0.245, *n* = 15, *p* = 0.378, ns
*Pan paniscus* males	*r*_*s*_ = 0.100, *n* = 9, *p* = 0.798, ns
*Pan paniscus* females	*r*_*s*_ = 0.115, *n* = 15, *p* = 0.684, ns

*Note*: Results presented in bold are statistically significant.

Abbreviation: ns, not significant.

Analyses to assess interpopulation differences in fWHR expression show that *Pan paniscus* have significantly higher fWHRs than do either *Pan troglodytes* subspecies (comparisons among three *Pan* groups: H_(2)_ = 15.304, *p* < 0.001; *Pan troglodytes* vs. *Pan paniscus* comparison: H_(1)_ = 14.181, *p* < 0.001, Figure 3). There were no significant fWHR differences between *Pan t. schweinfurthii* and *Pan t. troglodytes* (H_(1)_ = 1.152, *p* = 0.283). These findings indicate that selection for increased fWHR in *Pan paniscus* may have occurred since their split with *Pan troglodytes* and are consistent with the finding of an inverse relationship between fWHR and CCH in *Pan paniscus*. These findings further suggest that there is an increased emphasis on relatively wide faces, relative to CCH, in *Pan paniscus* for which average male and female crown height values are low relative to *Pan t. schweinfurthii* and *Pan t. troglodytes*, and where *Pan paniscus* show the lowest level of CCH dimorphism of all three *Pan* taxa (Table 5, Figure 4).

## 4 DISCUSSION

Overall, our findings are consistent with Weston et al.'s (2004) hypothesis that wide faces can evolve in species with smaller canine crowns. However, our results show that this pattern is not universal among *Pan* groups, where only *Pan paniscus* males show an inverse relationship between fWHR and CCH. Contrary to our expectations, *Pan t. troglodytes* show a positive association between these same two variables (wide faces are associated with *larger* canines). Together, our results suggest that among *Pan*, the mid‐face does not consistently replace CCH as a visual signal of aggression. Our findings that both chimpanzees and bonobos show fWHR sexual size dimorphism is consistent with findings of fWHR sexual dimorphism in *Pan paniscus* (Martin et al., 2019), though differs from previous research that shows the absence of fWHR sexual dimorphism in *Pan troglodytes* (Wilson et al., 2020). We further show that wide faces in *Pan t. schweinfurthii* males are associated with smaller body mass, suggesting that in addition to the inverse association between fWHR and CCH in this group, wide faces in *Pan t. schweinfurthii* males, but not in females, may be further driven by body size constraints. Our results suggest that there has been increased selection for wide faces in male and female bonobos, who have small canine teeth and wide faces, relative to *Pan troglodytes*, and have significantly higher fWHRs than do either chimpanzee subspecies.

Why might only one *Pan* taxon show an inverse relationship between fWHR and CCH? We suggest that under conditions where CCH is constrained in either sex (e.g., as a result of selection for increased jaw‐muscle leverage; Scott, 2010) or has lost its unique social signaling function, mid‐facial morphology through the expression of relatively wide faces may sometimes, but not always, be more heavily relied upon as an indicator of visual communication. This conclusion seems warranted given the large body of evidence to show that among modern humans and non‐human primates, wide faces are associated with personality and behavioral variables, including perception of aggression in modern humans (Sell et al., 2009; Stirrat & Perrett, 2010; Alrajih & Ward, 2014; Lefevre, Etchells, et al., 2014; Lefevre, Wilson, et al., 2014; Mileva et al., 2014; Zilioli et al., 2015; Anderl et al., 2016; Ormiston et al., 2017; Yang et al., 2018; Martin et al., 2019; Kajonius & Eldblom, 2020; Wen & Zheng, 2020; Wilson et al., 2020; Merlhiot et al., 2021; Brown et al., 2022; Wilson & Masilkova, 2023), and that canine teeth serve as a socially or sexually selected trait, used for visual signaling and weaponry (Leutenegger & Kelly, 1977; Plavcan, 1998; Plavcan, 2004; Plavcan et al., 1995; Plavcan & van Schaik, 1992, 1993). This may be in the context of sexual or social selection (e.g., Haselhuhn et al., 2015; Hodges‐Simeon et al., 2021; Lefevre, Etchells, et al., 2014), where wide faces may serve as an honest visual signal, in lieu of large canines, of an individual's potential to engage in physical aggression, anger, threat and dominance behavior (Weston et al., 2004).

The findings of differing relationships between fWHR, and CCH and body mass, respectively, in *Pan t. schweinfurthii* and *Pan t. troglodytes* are intriguing in that *Pan t. schweinfurthii* males show a significant association between fWHR and body mass, which is not found in either sex of *Pan t. troglodytes*. Further, our findings show that there is a positive relationship between fWHR and CCH in *Pan t. troglodytes*, which is not observed in *Pan t. schweinfurthii* and *Pan paniscus*. *Pan t. schweinfurthii* are the smallest of the chimpanzee subspecies (*Pan t. schweinfurthii* male mean body weight 42.7 kg, female mean body weight 33.7 kg) (Smith & Jungers, 1997; Uehara & Nishida, 1987), which is similar to *Pan paniscus* body weight (*Pan paniscus* male mean body weight 45.0 kg, female mean body weight 33.2 kg) (Jungers & Susman, 1984). By contrast, *Pan t. troglodytes* males and females are approx. 35%–40% larger than the other two *Pan* taxa considered in this study (*Pan t. troglodytes* male mean body weight 59.7 kg, female mean body weight 45.8 kg) (Jungers & Susman, 1984; Smith & Jungers, 1997). *Pan t. schweinfurthii* and *Pan paniscus* live in close geographical proximity to one another and are also similar in that they have a relatively heavy reliance on terrestrial herbaceous vegetation (THV) as fallback foods during periods of preferred‐food scarcity compared to *Pan t. troglodytes* (Furuichi et al., 2001; Tutin et al., 1997; Wrangham et al., 1996). This is consistent with the smaller body size in *Pan t. schweinfurthii* and *Pan paniscus*, compared with *Pan t. troglodytes* (Smith & Jungers, 1997). Smaller body size may be selected for among *Pan t. schweinfurthii* and *Pan paniscus* due to a reduced energy turnover, associated with periods of reduced metabolizable energy during periods of preferred‐food scarcity (e.g., Simmen et al., 2017). Selective pressures associated with small body size among *Pan t. schweinfurthii* and *Pan paniscus*, potentially driven by ecological constraints, may mean that relative facial breadth takes on an increased social signaling function that would otherwise be communicated by larger body size among males.

Socioecological differences among *Pan troglodytes* subspecies and *Pan paniscus* may be associated with how body mass and CCH are associated with fWHR variation among groups. Chimpanzees and bonobos vary in their patterns of social grouping, where some taxa live in male‐bonded systems in which only males exhibit territorial behavior, while others live in bisexually‐bonded social systems in which both males and females use their home range equally, and both sexes contribute to the group's ability to compete and actively participate in maintaining territorial boundaries (Boesch et al., 2008; Lemoine et al., 2020; Williams et al., 2004). Among bisexually‐bonded chimpanzee groups, group size is critical in maintaining territory boundaries (Lemoine et al., 2020). These findings suggest that male ability to defend territory boundaries may be less important among bisexually‐bonded groups than among male‐bonded groups. It is therefore likely that among bisexually‐bonded groups, that is, when group size and coalitionary behavior plays a large role in territorial defense, selection on physical traits associated with lethal intergroup aggression may be relaxed (Plavcan, 2004; Plavcan et al., 1995). This is also hypothesized to be the case among bonobos, who do not show intergroup lethal aggression (Gruber & Clay, 2016). Interpopulation differences in tradeoff patterns among fWHR, CCH and body size, as has been observed in the present study, may therefore be associated with selection patterns for these traits in the context of how they are used for visual signaling and combat ability based on the frequency of intragroup versus intergroup aggression across *Pan* groups. Specifically, populations for whom rates of lethal intergroup aggression among males are high may be subject to more stringent selective pressures for morphological traits that are associated with winning combative encounters (e.g., large canine teeth), compared with groups that rarely engage in intergroup conflict. This hypothesis requires further testing, and further investigations into patterns of fWHR and CCH variation, in the context of interspecific differences in socioecological variables, are likely to provide further insights as to what may be driving the expression of wide faces in *Pan* males and females.

The finding that the relationships between fWHR and CCH are not universal among *Pan* groups mirrors the lack of universality in the relationships among fWHR and personality or behavioral variables, as well as interpopulation differences in the presence and pattern of fWHR sexual dimorphism expression among human populations and non‐human primate taxa. Among humans, chimpanzees and bonobos alike, it is currently unclear whether the interpopulation variation in the associations between morphological and personality or behavioral variables is because populations are undergoing different selective pressures in the context of the localized socioecological modern environment. It is also unclear among humans alone, whether relatively wide faces have lost their signaling function in the context of an evolutionary mismatch between the visual signaling function of fWHR and any corresponding potential for aggressive behavior (Durkee & Ayers, 2021; Wang et al., 2019). The finding that fWHR sexual dimorphism persists in some modern human and extant primate groups (Caton & Dixson, 2022; Kramer, 2017; Lefevre et al., 2012; Özener, 2012; Rostovtseva et al., 2021; Summersby et al., 2022; Weston et al., 2007), even when controlling for facial height, craniometric and body size measurements (e.g., Caton & Dixson, 2022) indicates that relative facial breadth may continue to serve a visual signaling function (cf. Durkee & Ayers, 2021; Wang et al., 2019). Among a large sample of Australian citizens of mixed ethnicity, older females (i.e., those over the age of 48 years old) show larger fWHR values than males, in contrast to male‐biased sexual dimorphism observed in young adulthood (Summersby et al., 2022). This finding, along with those of female‐biased sexual dimorphism among Buryats of Southern Siberia and Caucasian young adults (Lefevre et al., 2012; Rostovtseva et al., 2021) provide further evidence to suggest that wide faces may serve a social signaling function in females as well as males.

Our findings of fWHR sexual dimorphism in *Pan troglodytes* contrast with those by Wilson et al. (2020), who found no fWHR dimorphism in *Pan t. schweinfurthii*, *Pan t. troglodytes* or *Pan t. verus*. Possible explanations for this discrepancy may be associated with methodological and sampling differences. Our study used 3D surface models of dry cranial specimens to measure fWHR, whereas Wilson et al. (2020) used 2D photographs of living chimpanzees to collect fWHR data, which may be a less accurate measure of true facial height, given that *Pan* taxa show substantial facial prognathism. The 2D photographs used by Wilson et al. (2020) also contained soft tissue information that overlays the bony hard tissue, meaning that measurements derived from photographs of living chimpanzees may incoporate this additional source of soft tissue variation, which has the potential to vary across the adult lifespan in non‐human primates (e.g., Paukner et al., 2021). The source of each sample may alternatively account for differences in findings, where our *Pan troglodytes* sample was obtained from wild individuals of known locality, whereas those used in Wilson et al.'s (2020) study were obtained from captive chimpanzees from zoos in several geographical locations (United States, United Kingdom and Japan), across 15 facilities, who likely experienced substantial variation in living conditions and opportunities for social interactions. It is also possible that the age range of specimens in our sample may account for differences in our study, compared with that of Wilson et al. (2020). As the specimens in our sample were included on the basis that they showed relatively unworn canine dentition, our sample likely represents young to middle‐aged adults, with the oldest specimens being excluded from our sample. Wilson et al.'s (2020) study included individuals up to the age of 49 years, and it is likely that age‐related sampling may partially explain the discrepancy in findings. This is particularly plausible given that age‐related craniofacial changes beyond dental maturity have been observed among humans and non‐human primates, including those associated with craniofacial breadth and fWHR (Balolia et al., 2013, 2017; Balolia & Fitzgerald, 2024; Barel Hooge et al., 2024; Hodges‐Simeon et al., 2021; Lefevre, Wilson, et al., 2014; Martin et al., 2019; Wang et al., 2007).

An outstanding question relates to the mechanism that is driving interpopulation differences in the associations between fWHR, CCH and body mass among taxa and between sexes. Evidence suggests that increased relative facial breadth is a sexually or socially selected trait, either through intrasexual signaling of an ability to succeed in competitive encounters, or as a result of mate choice (e.g., Hodges‐Simeon et al., 2021; Weston et al., 2007). As previously noted, sex differences in fWHR emerge in early or mid‐adulthood in some non‐human primate taxa and human populations (Hodges‐Simeon et al., 2021; Lefevre, Wilson, et al., 2014; Wilson et al., 2014). Among another modern human group, younger males show larger fWHR values than older males (Summersby et al., 2022). Similarly, among capuchin monkeys, age only has a significant effect on male fWHR in the context of developmental status when the oldest (presumably non‐reproducing) individuals are excluded from the sample (Lefevre, Wilson, et al., 2014), indicating that the signaling quality of relatively wide faces is only relevant among reproductively active individuals. Increases in body weight and crown‐rump length among male and female chimpanzees in adolescence is associated with rapid increases in testicular size and circulating inhibin (males only) and circulating testosterone in both sexes (Copeland et al., 1985; Marson et al., 1991; Hamada et al., 1996). Further, there are interspecific differences in puberty onset among chimpanzees and bonobos, especially among females, which is mediated by testosterone levels (Behringer et al., 2014). While there is evidence of hormonal changes around the time of puberty in chimpanzees and bonobos, it is currently unclear how hormonal differences may be associated with variation in fWHR among *Pan*. It is possible that interpopulation differences in the development and expression of increased relative facial breadth in early adulthood in *Pan* are associated with hormonal changes in the context of a socially selected trait which serves as a signal of social maturity, and the ability to successfully engage in competitive encounters. In addition, the extent to which interspecific differences in the degree of facial projection/prognathism impacts fWHR values and associated interpretations has yet to be thoroughly investigated, especially in the context of the relatively low degree of facial prognathism found among modern humans, relative to chimpanzees, bonobos and other primate species (Bastir & Rosas, 2004). Therefore, the influence of variation associated with facial prognathism ought to be taken into consideration when comparing fWHR values among species that show facial projection differences.

Among humans, there is evidence to suggest that raters can accurately assess body strength, fighting ability and physical threat potential based on observations of facial characteristics (MacDonell et al., 2018; Sell et al., 2009; Zilioli et al., 2015). If fWHR is a valid indicator of aggression potential and fighting ability then it is surprising that a statistically significant negative relationship between fWHR and body mass estimates (which are likely associated with physical strength) exists in one chimpanzee population (*Pan t. schweinfurthii*), as reported in this study. It is also unclear as to why only one of the three *Pan* groups investigated as part of this study shows this relationship. However, the fact that only one *Pan* groups shows this association (i.e., that the relationship between body size and fWHR is not universal across populations) allows scope for further investigations in *Pan* spp. and *Homo sapiens* alike to understand what behavioral or personality variables, and ecological or socioecological conditions are associated with variation in fWHR expression in the context of body size. This will afford a better understanding of whether fWHR is a trait that is used in visual communication to maximize fitness among modern humans, or whether there is an evolutionary mismatch surrounding the retention of perception biases associated with relative facial breadth that is no longer functionally relevant in societies today.

## AUTHOR CONTRIBUTIONS

**Katharine L. Balolia:** Conceptualization (lead); formal analysis (lead); methodology (equal); visualization (lead); writing – original draft (lead); writing – review and editing (lead). **Kieran Baughan:** Investigation (lead); methodology (equal); writing – original draft (supporting); writing – review and editing (supporting). **Jason S. Massey:** Methodology (equal); resources (lead); writing – review and editing (supporting).

## FUNDING INFORMATION

This research did not receive any specific grant from funding agencies in the public, commercial, or not‐for‐profit sectors.

## CONFLICT OF INTEREST STATEMENT

The authors declare no conflict of interest.

## Data Availability

The data that support the findings of this study are available via Figshare: https://doi.org/10.6084/m9.figshare.24549649.
